# Psychometric properties of the modified Drug Abuse Screening Test Sinhala version (DAST-SL): evaluation of reliability and validity in Sri Lanka

**DOI:** 10.1186/s12889-024-19288-x

**Published:** 2024-07-03

**Authors:** Sashiprabha Dulanjalee Nawaratne, Janaki Vidanapathirana

**Affiliations:** 1 Directorate of Healthcare Quality and Safety, Ministry of Health, Premises of Castle Street Hospital for Women, Colombo, 08 Sri Lanka; 2 Planning Unit, Ministry of Health, No. 385, Rev. Baddegama Wimalawansa Thero Mawatha, Colombo, 10 Sri Lanka

**Keywords:** Drug abuse screening test, Psychometric properties, Validation study, Sri Lanka

## Abstract

**Background:**

Psychoactive drug use is an important public health issue in Sri Lanka as it causes substantial health, social and economic burden to the country. Screening for substance use disorders in people who use drugs is vital in preventive health care, as it can help to identify problematic use early. Screening can aid in referring those in need, for the most appropriate treatment and care. Thus, preventing them from developing severe substance use disorders with complications. The Drug Abuse Screening Test (DAST-10) is an evidence-based tool widely used to assess the severity of psychoactive drug use. This study aimed to culturally adapt and evaluate the validity and reliability of the Drug Abuse Screening Test (DAST-10) in Sri Lanka.

**Methods:**

The DAST-10 was culturally adapted, and the nine-item Sinhala version (DAST-SL) was validated using exploratory and confirmatory factor analysis. The validation study was conducted in the Kandy district among people who use drugs, recruited using respondent-driven sampling. Criterion validity of the questionnaire was assessed by taking the diagnosis by a psychiatrist as the gold standard. Cut-off values for the modified questionnaire were developed by constructing Receiver Operating Characteristic (ROC) curves. The reliability of the DAST-SL was assessed by measuring its internal consistency and test re-test reliability.

**Results:**

The validated DAST-SL demonstrated a one-factor model. A cut-off value of ≥ 2 demonstrated the presence of substance use disorder and had a sensitivity of 98.7%, specificity of 91.7%, a positive predictive value of 98.8% and a negative predictive value of 91.3%. The area under the curve of the ROC curve was 0.98. A cut-off score of ≤ 1 was considered a low level of problems associated with drug use. The DAST-SL score of 2–3 demonstrated a moderate level of problem severity, a score of 4–6 demonstrated a substantial level of problems, and a score of ≥ 7 demonstrated a severe level of drug-related problems. The questionnaire demonstrated high reliability with an internal consistency of 0.80 determined by Kuder–Richardson Formula-20 and an inter-class correlation coefficient of 0.97 for test re-test reliability.

**Conclusion:**

The DAST-SL questionnaire is a valid and reliable tool to screen for drug use problem severity in people who use drugs in Sri Lanka.

**Supplementary Information:**

The online version contains supplementary material available at 10.1186/s12889-024-19288-x.

## Introduction

Psychoactive drug use affects every aspect of society, from social systems to people alike [1]. Psychoactive drugs are defined as “substances that, when taken, have the ability to change an individual’s consciousness, mood or thinking processes” [2]. Substance use disorders resulting from psychoactive drug use cause adverse impacts not only on the health of the individual, but also on the economy, culture, and development of a country [3]. Due to its proximity to both the “golden triangle” (Thailand, Myanmar, and Vietnam) and the “golden crescent” (Afghanistan, Iran, and Pakistan), the regional and international drug problem has readily found its way to Sri Lanka [4]. The prevalence of psychoactive drug use in a country can be identified through different methods, such as the number and the trends of arrests [5] and Sri Lanka has seen a steady increase in people who use drugs over the years [6]. Total drug-related arrests increased by 26% in just half a decade with 110,031 arrests in 2021 [7] compared to the 79,378 arrests reported in 2016 [8]. According to the National Prevalence Survey 2019, there are an estimated 533,883 people who use drugs in Sri Lanka [9].

In Sri Lanka, treatment for substance use disorders is provided by both the health and non-health sectors. Hospital-based outpatient clinics led by psychiatrists and community-based outreach clinics conducted by medical officers under the supervision of district psychiatrists provide services in the health sector [10]. In the non-health sector, the National Dangerous Drugs Control Board, non-government organizations and the rehabilitation centre of the Bureau of Commissioner General of Rehabilitation provide treatment services [11]. However, according to national survey data, only 7% of the total people who use drugs in Sri Lanka access treatment services yearly [9].

The use of psychoactive drugs can impair a person’s sleep and nutrition, as well as increase their risk of trauma, violence, injury, and contracting infectious diseases including HIV/AIDS and Hepatitis C [12]. Therefore, people who use drugs are encountered at many other service delivery points in Sri Lanka, such as hospital outpatient departments (OPD), primary health care institutions, general practitioners, accident and emergency departments in hospitals, Sexually Transmitted Disease (STD) clinics in hospitals, and non-governmental organisations working with drug users. However, most services are not utilised as referral points for treatment due to the difficulty in identifying people in need of treatment and the lack of time for assessment.

To ensure good clinical care for people who use drugs, it is necessary to assess the level of their psychoactive drug use severity [13]. The inability to capture drug use problems in the early stages may also result in them seeking treatment when they develop severe substance use disorder (SUD) with complications [13]. Screening, which can be used in a variety of settings, will not only provide reliable and valid information to the care provider but will also help refer them to suitable treatment services for tailored interventions. Furthermore, early initiation of treatment through screening not only improves the overall quality of life of people who use drugs but is also proven to be cost-effective to the country’s economy as it reduces health care and criminal justice costs [12].

## Drug Abuse Screening Test (DAST)

The original Drug Abuse Screening Test (DAST), developed by Professor Harvey Skinner, included 28 items that assess the severity of psychoactive drug use [14]. Two shortened versions were developed later with 20 and 10 items with outstanding internal consistency and reliability for such a short scale [15]. The DAST-20 provides a broader evaluation and is more suitable for clinical assessment, while the DAST-10 is more suitable for case finding in outpatient and non-clinical settings [15]. Therefore, we selected the DAST-10 for our study.

The DAST 10 uses ‘yes’ or ‘no’ type answers to assess the severity of psychoactive drug use or problems related to psychoactive drug use of an individual [15]. Due to its nature, the DAST-10 is easy to administer with relatively little to no training in a variety of settings [15]. It has been used among patients with mental illnesses [16, 17], prisoners [18], patients receiving treatment for psychoactive drug use problems [19, 20], burn patients [21], pregnant women [22], and the general adult population [23, 24]. The tool has been assessed for its reliability and validity in various settings in different countries and has proven to have good psychometric properties [25]. The DAST-10 has been validated in different languages to screen for problems associated with psychoactive substances other than alcohol and tobacco [15]. It has demonstrated good internal consistency with Cronbach’s alpha ranging from 0.71-0.94 [19, 24, 26–29]; good test re-test reliability with correlation coefficient ranging from 0.71–0.85 [26, 27]; high sensitivity varying between 79.2%-98% and specificity ranging between 67.7%-96.2% [19, 20, 22, 26]. Factor analysis of the DAST-10 has demonstrated good construct validity. A few studies suggest that the DAST-10 has a three-dimensional factor structure [22, 29] while the majority of studies suggest a unidimensional structure [19, 20, 24, 28].

The DAST-10 has never been validated in Sri Lanka, nor has it been validated elsewhere to assess the level of psychoactive drug use among non-institutionalised people who use drugs. Our study aimed to culturally adapt and evaluate the validity and reliability of the Sinhala version of the Drug Abuse Screening Test questionnaire among people who use drugs in Sri Lanka.

## Methods

### Cultural adaptation

A cross-cultural adaptation process is required when an instrument is applied in a different culture, language or country from the country of its origin [30]. For cultural adaptation, we used the method described by Beaton et al. [30] with a modified Delphi technique employing an expert review. The Delphi technique emphasises systematic anonymous communication among people with expertise in a certain topic with the aim of reaching a consensus [31]. We used a modified Delphi technique, in which the experts in the panel were anonymous to each other but not to the investigators. This approach helped the investigators to maintain better communication with the panellists and work around their busy schedules. The expert panel consisted of four consultant psychiatrists, two psychologists, a sociologist, and two consultant community physicians.

The modified Delphi technique was used to get the consensus of the experts in the pre-and post-translation stages. In the pre-translation stage, a consensus was reached for items in the DAST-10 on the appropriateness of wording used, cultural acceptability and suitability for use in the local context. The panellists reviewed each item of the tool and rated the items on a 4-point Likert scale, with ‘1’ being the least acceptable and ‘4’ being the most acceptable. If more than 75% of the panellists rated an item as ‘4’ on the Likert scale, it was considered that consensus was reached for that item. After the pre-translation expert panel review, the first item of the DAST-10 was removed. The changes were communicated with the original author and the DAST version 1.0 was translated.

**Fig. 1:**
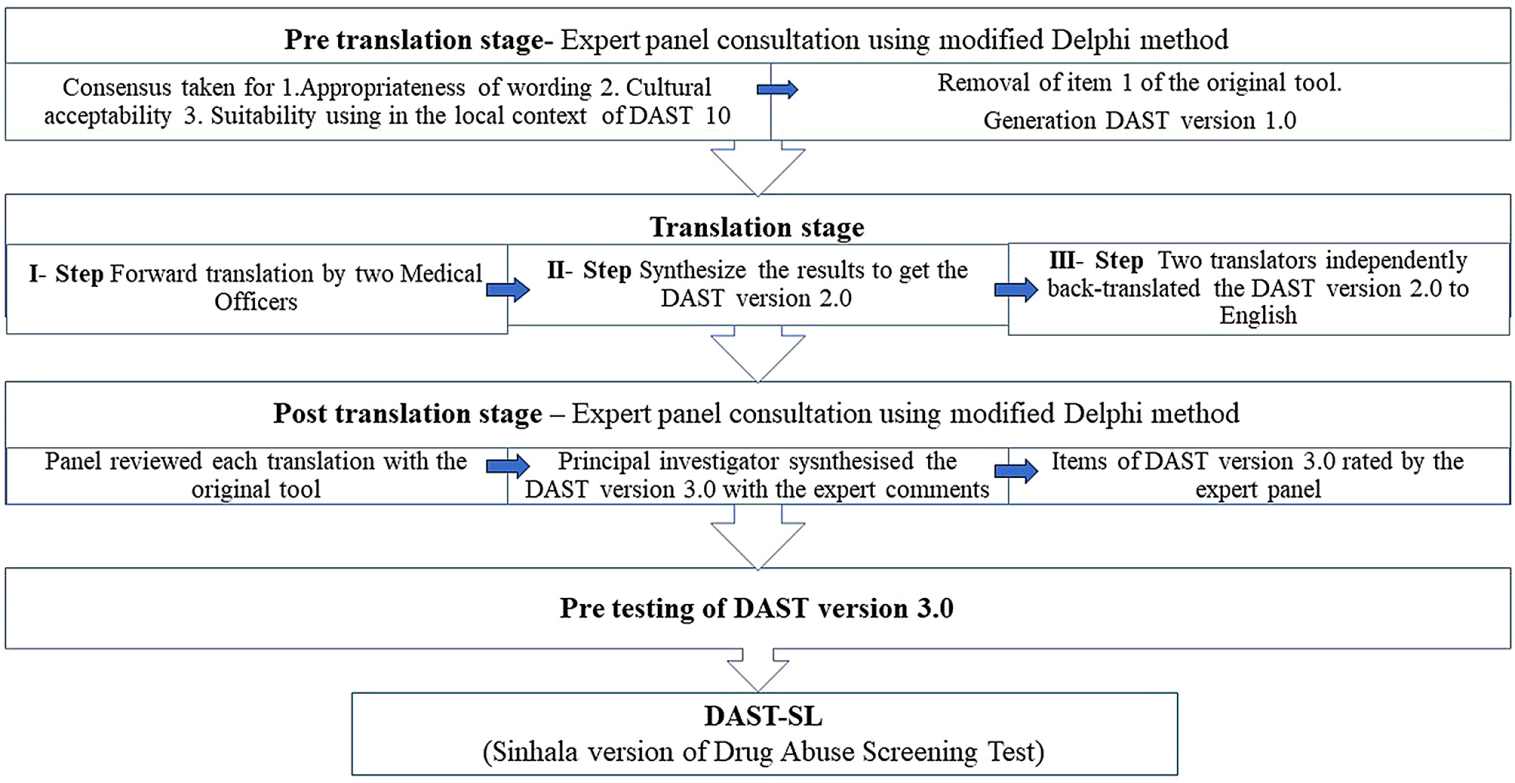
Cultural adaptation process of the Drug Abuse Screening Test questionnaire

During the translation stage of the DAST version 1.0, the process described by Beaton et al. was followed [30]. The forward translation was carried out as the first step by two medical practitioners who were fluent in both English and Sinhala. The principal investigator then got down with the two translators and synthesised the results to create DAST version 2.0. In the third step, one bilingual medical professional and one bilingual translator, both of whom were blinded to the original English version of the DAST-10, independently back-translated the DAST version 2.0 to English. This was done to check for any gross inconsistencies or conceptual errors that occurred during the translation [30]. At the post-translation stage, the expert panel then reviewed each translation with the original tool, to assess the comparability of meaning and general linguistic clarity. The principal investigator with the expert comments then synthesised the DAST version 3.0. The expert panel reviewed the DAST version 3.0, rating each item on a 5-point Likert scale (“strongly disagree”, “disagree”, “undecided”, “agree”, “strongly agree”). If more than 75% of the expert panellists responded “agree” or “strongly agree” on a particular item, it was decided that consensus had been reached. This cut-off (> 75%) was thought to indicate widespread agreement among the significant majority of the participants [32]. The level of agreement was predetermined. The DAST version 3.0 underwent two rounds of Delphi and the pre-final DAST-SL was developed. To further evaluate the appropriateness of the content and the clarity of the expression, pre-testing of the DAST-SL was performed among 10 people who use drugs attending the substance use clinic at the National Hospital-Kandy, Sri Lanka. The key steps of the cultural adaptation process are illustrated in Fig. 1.

### Content validity

Content validity evaluates whether the content of the tool covers the relevant and necessary areas of the subject it intends to measure [33]. The content validity of the DAST-SL was assessed by a panel of seven experts from different disciplines (community medicine, psychiatry, clinical psychology, and sociology). These experts were not involved in the cultural adaptation process of the DAST 10 questionnaire. Each panellist was provided with the finalised DAST-SL questionnaire. The experts assessed the relevance of each item of the tool for assessing the severity of psychoactive drug use among people who use drugs in Sri Lanka. Each panellist rated each item for relevance on a 4-point Likert scale (1- not relevant, 2- relevant but needs major revision, 3- relevant but needs minor revision, 4- very relevant). To calculate the average level of agreement, the procedure described by Zamanzadeh et al. was used [34]. First, the responses were amalgamated into two categories by combining 1 and 2 as “not relevant” and 3 and 4 as “relevant”. Each response carried one mark. Therefore, the total marks for being “relevant” or “not relevant” were calculated for each item. Then for each item, the total score for “relevant” was divided by the number of experts and converted to a percentage. An item score of 80% or higher among the experts was considered appropriate [34]. During content validity, no item of the DAST-SL was rated as inappropriate or not relevant.

### Construct validity

During cultural adaptation, the first item was removed from the questionnaire. Therefore, Exploratory Factor Analysis (EFA) was conducted to identify the possible underlying factor structure of the nine-item Drug Abuse Screening Test-Sri Lanka (DAST-SL). Since more observations per item ensure more stable results [35], the item-to-observation ratio was taken as 1:20, and 180 people who use drugs, > 18 years of age, who used psychoactive drugs for nonmedical purposes for any duration during the 12 months preceding the study, who possessed a valid recruitment coupon, who resided in the Kandy Municipal area were recruited using the Respondent-Driven Sampling (RDS) method. RDS is a type of chain referral sampling method used to recruit hard-to-reach population groups [36]. To enable the recruitment of peers through peers, the RDS uses coupons that carry a unique identification number, purpose of the study, days and time of data collection, expiration date, and contact information of the investigator [37]. The unique identification number in the coupons helps to visualize the recruitment pattern of the participants, as shown in Additional file 1. Each participant was issued three recruitment coupons and asked to pass them on to their peers. The principal investigator administered the questionnaire to all participants who fulfilled the inclusion criteria and provided consent. Eigenvalues and a scree plot diagram were used to assess the EFA.

Confirmatory Factor Analysis (CFA) was also conducted to see how well the factor structure observed by the EFA was replicated by the data of the study [38]. Model fit indices were evaluated to select the best model [38]. Mplus software version 8.4 was used to perform both the EFA and the CFA [39]. A questionnaire containing basic socio-economic information, drug use patterns, and the DAST-SL was developed for data collection. The English version of the questionnaire used for assessing the construct validity is shown in Additional file 2.

### Criterion validity

To assess criterion validity, the DAST-SL was administered to the participants by the principal investigator before the interview with a psychiatrist for clinical assessment. All participants in the study understood and communicated in Sinhala well. The severity levels (mild, moderate and severe) of SUD were assessed according to the Diagnostic and Statistical Manual of Mental Disorders, Fifth Edition (DSM-5) classification for SUD [40], which was provided by a consultant psychiatrist. The DSM criteria were chosen for better comparability, as the DAST has been validated against DSM criteria [41], and different adaptations of the tool have used correlations with DSM criteria to assess its validity [19, 20, 42]. The consultant psychiatrist who conducted the clinical interviews was well-versed in the DSM-5 criteria for substance use disorder (SUD) and had a special interest in psychoactive drug use. The participants who were diagnosed with SUD by the psychiatrist were provided with a referral letter to an institution of their choosing (hospital clinic or centre conducted by the National Dangerous Drugs Control Board). The scores generated by the DAST-SL and clinical diagnosis of SUD were compared. To determine the clinically validated cut-off points for psychoactive drug use severity, Receiver Operating Characteristic (ROC) curves were generated using Statistical Package for the Social Sciences (SPSS) version 22. The optimal cut-off scores were developed by constructing the following ROC curves; (a) not having a SUD vs. having a SUD, (b) not having SUD and mild SUD vs. moderate SUD and severe SUD, and (c) not having SUD, mild SUD and moderate SUD vs. severe SUD. The best cut-off point is taken as the point that had the highest true positive rate and the lowest false positive rate. To determine the optimal cut-off values, the Youden index was calculated [43].

The minimum sample size required to assess the criterion validity of the tool was also used for the CFA. Sample size calculation was based on the expected sensitivity or specificity of the instrument, the required level of precision and the confidence interval [44]. For CFA and criterion validation, 183 people who use drugs were recruited from the Kandy District, using RDS excluding the Kandy municipal area, using the same inclusion criteria as those used in EFA. Sampling was initiated with six seeds, with a recruitment quota of three. The participant recruitment trees generated using the RDS-Analyst software are shown in Additional file 3.

### Reliability

Reliability was assessed by checking for internal consistency and test re-test reliability. The Kuder–Richardson Formula-20 (KR-20), the nonparametric equivalent of Cronbach’s alpha, was used to assess internal consistency. Internal consistency was used to check if the nine items of the DAST-SL were measuring the same construct [45]. The KR-20 was selected because the item responses on the DAST-SL were dichotomously scored, and it is a good measure for estimating internal consistency with binary variables [45]. The test re-test reliability was measured by calculating the interclass correlation coefficient. Test re-test reliability assesses the stability of the construct of the tool with time [46]. The test re-test reliability was measured by administering the DAST-SL to a subsample of 20 people who use drugs, following a time interval of 10–14 days. The interclass correlation coefficient was calculated by correlating the DAST-SL scores for the first time with the scores obtained on the second time, after 10–14 days [46]. The internal consistency and test re-test reliability were calculated using the Statistical Package for the Social Sciences (SPSS) version 22.

Data collection was carried out by the principal investigator from the 29th of July to the 13th of September 2019. Informed written consent was obtained from all participants before administering the questionnaires. Administration approval for the study was obtained from the Provincial Director of Health Services, Central Province, Sri Lanka, and ethical approval was obtained from the Ethical Review Committee of the Faculty of Medicine, University of Colombo (EC-19-055).

## Results

### Cultural adaptation

During the cultural adaptation, none of the items were identified as culturally inappropriate. However, the first question “Have you used drugs other than those required for medical reasons?” was suggested as not relevant as the inclusion criteria for the study included only people who use drugs. No new items were suggested to be added to the questionnaire by the experts. The experts suggest modifying item numbers ‘eight’ and ‘nine’ by including examples to clarify ‘illegal activities’ and ‘withdrawal symptoms’. The final DAST-SL was a brief nine-item tool with ‘yes’ or ‘no’ answers. Each question answered as ‘yes’ was given a score of one and each question answered as ‘no’ was given zero marks except for item number two. Item number two was scored reversely. Therefore, the total score on the DAST-SL ranged from zero to nine.

### Exploratory and confirmatory factor analysis

For the validation study, all participants who possessed a valid coupon that came to the data collection site completed the questionnaire. Hence, there were no non-responses. The socio-demographic characteristics and psychoactive drug use patterns of the participants are shown in Table 1.

**Table 1:** Distribution of the study population for the exploratory factor analysis by their socio-demographic characteristics and psychoactive drug use patterns

Characteristics		Frequency (*n*=180)	Percentage (%)
Age category	<20 years	10	5.55
	21-30 years	36	20.00
	31-40 years	85	47.22
	41-50 years	32	17.78
	51-60 years	12	6.67
	>61 years	5	2.78
Sex	Female	2	1.11
	Male	178	98.89
Ethnicity	Sinhala	159	88.33
	Tamil	19	10.56
	Muslim	2	1.11
Highest level of education	Never attended school	2	1.11
	Grade 1-5	25	13.89
	Grade 6-10	110	61.11
	Passed General Certificate of Education (GCE) Ordinary Level	29	16.11
	Grade 12-13	11	6.11
	General Certificate of Education (GCE)/Advanced Level and above	3	1.67
Current Employment	Unemployed	18	10.00
	Employed	162	90.00
Monthly income	< Rs.20,000	18	10.00
	Rs. 20,001 – 40,000	88	48.89
	Rs. 40,001 – 60,000	43	23.89
	Rs. 60,001 – 80,000	15	8.33
	Rs. 80,001 – 100,000	10	5.56
	> Rs. 100,001	6	3.33
Type of drugs used*	Cannabis	134	74.44
	Heroin	95	52.78
	Pregabalin	43	23.89
	Tramadol	42	23.33
	Methamphetamine	38	21.11
	Diazepam	26	14.44
	Others	9	5.00
Frequency of psychoactive	2 to 3 times a month or less	17	9.44
drug use	About once a week	20	11.11
	2 to 3 times a week	17	9.44
	About once a day	34	18.90
	2 times or more in a day	92	51.11

* Multiple responses

**Fig. 2:**
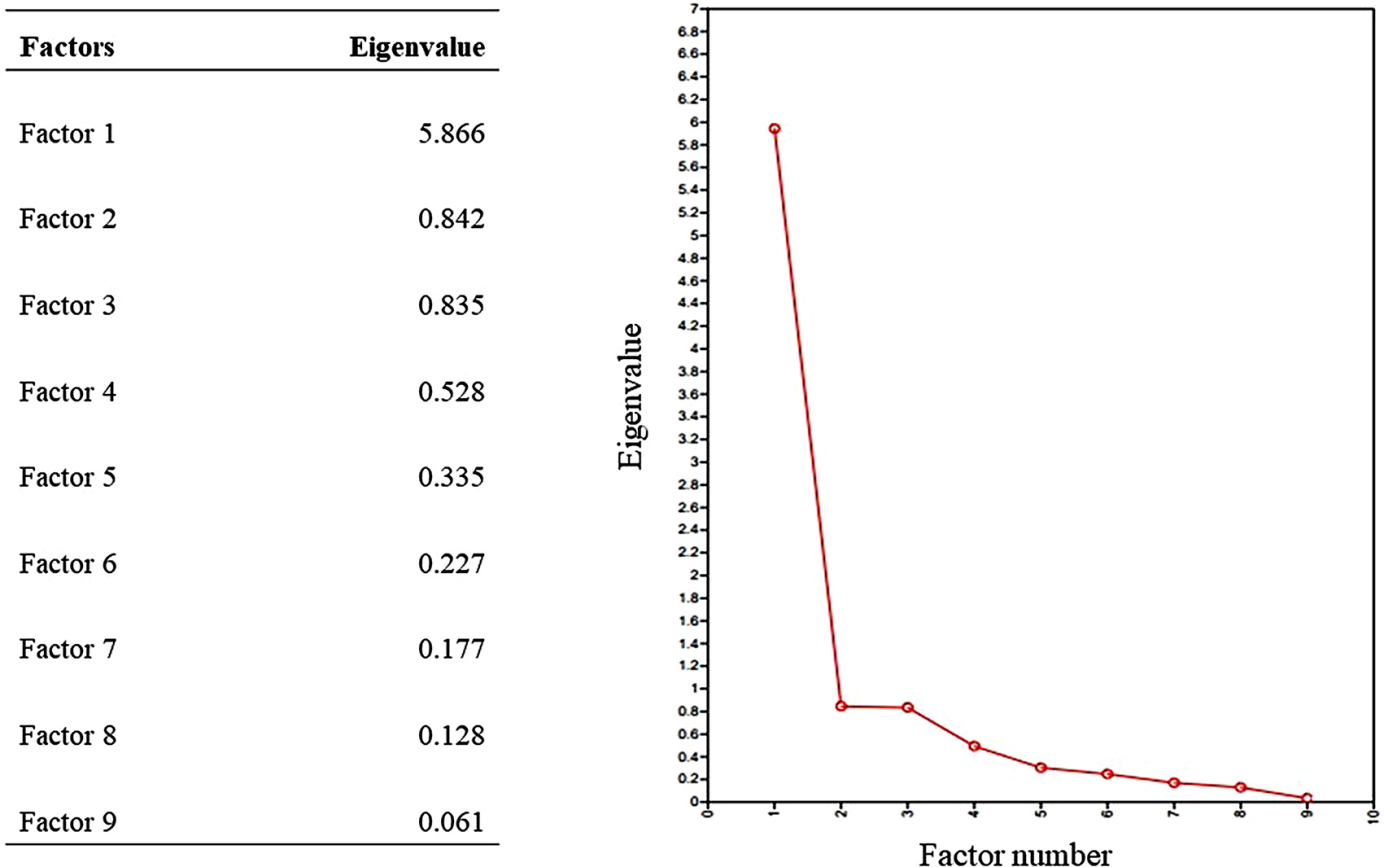
Eigenvalues and scree plot diagram for the Sinhala version of the Drug Abuse Screening Test

For EFA, an eigenvalue greater than one was observed only once. This suggested that the one-factor model for the DAST-SL was more appropriate. This was confirmed by visual inspection of the scree plot, as shown in Fig. 2.

The median age of the participants in the CFA was 38 years (ICR ± 20 years). Among the participants, the majority were male (*n* = 179;97.8%). Sinhala participants were 82.5%(*n* = 151), followed by Tamil (*n* = 30;16.3%) and Muslim (*n* = 2;1.1%). One per cent of the participants had never attended school, while the majority had education up to grade 6–10 (*n* = 123;67.2%). Eighty-eight per cent were currently employed, while 60% (*n* = 110) had elementary occupations. The majority had a monthly income of 20,001–40,000 Sri Lankan Rupees (*n* = 92;50.3%). Most participants used cannabis (*n* = 140;76.5%) followed by opioids (*n* = 113;61.7%) and 65% (*n* = 119) of participants used drugs at least once a day. The item responses for the DAST-SL given by the participants are depicted in Table 2.

**Table 2:** The modified drug abuse screening test questionnaire (DAST-SL) and its item responses

Item	Yes		No				
	No.	%	No.	%	Mean	SD*	σ²**
1. Do you abuse more than one drug at a time?	121	66.12	62	33.88	0.66	0.48	0.23
2. Are you always able to stop using drugs when you want to?	101	55.19	82	44.81	0.45	0.50	0.25
3. Have you had “blackouts” or “flashbacks” as a result of drug use?	75	40.98	108	59.02	0.41	0.49	0.24
4. Do you ever feel bad or guilty about your drug use?	129	70.49	54	29.51	0.70	0.46	0.21
5. Does your spouse (or parents) ever complain about your involvement with drugs?	140	76.50	43	23.50	0.77	0.43	0.18
6. Have you neglected your family because of your use of drugs?	96	52.46	87	47.54	0.52	0.50	0.25
7. Have you engaged in illegal activities in order to obtain drugs? (e.g. theft, fraud, prostitution)	70	38.25	113	61.75	0.38	0.48	0.24
8. Have you ever experienced withdrawal symptoms (felt sick) when you stopped taking drugs? (e.g. headaches, dizziness, chest tightness, difficulty breathing, nausea, vomiting, diarrhoea, stomach aches, tremors, muscle aches, sweating)	110	60.11	73	39.89	0.60	0.49	0.24
9. Have you had medical problems as a result of your drug use (e.g. memory loss, hepatitis, convulsions, bleeding, etc.)?	38	20.77	145	79.23	0.21	0.41	0.17

*Standard Deviation

** Variance

For CFA, the one-factor model demonstrated good goodness of fit indices with a Chi-square value (χ2) of 36.09, a p-value of 0.11, a Comparative Fit Index (CFI) of 0.993 and a Tucker-Lewis Index (TLI) of 0.991. The Root-Mean-Square Error of Approximation (RMSEA) was 0.043 which was less than the expected value of < 0.05. The Standardized Root Mean Square Residual value (SRMR) was 0.056.

During CFA, item three of the questionnaire demonstrated a poor correlation with the total score, as shown in Table 3. However, when it was removed from the questionnaire, the model demonstrated poor goodness of fit indices. Therefore, it was deemed justifiable to keep all the items of the questionnaire without removing item three.

**Table 3:** Item correlation of the Sinhala version of the modified drug abuse screening test questionnaire

Item number	Correlation
Item 1	0.507
Item 2	0.871
Item 3	0.219
Item 4	0.686
Item 5	0.812
Item 6	0.739
Item 7	0.641
Item 8	0.947
Item 9	0.921

### Assessing criterion validity

**Fig. 3:**
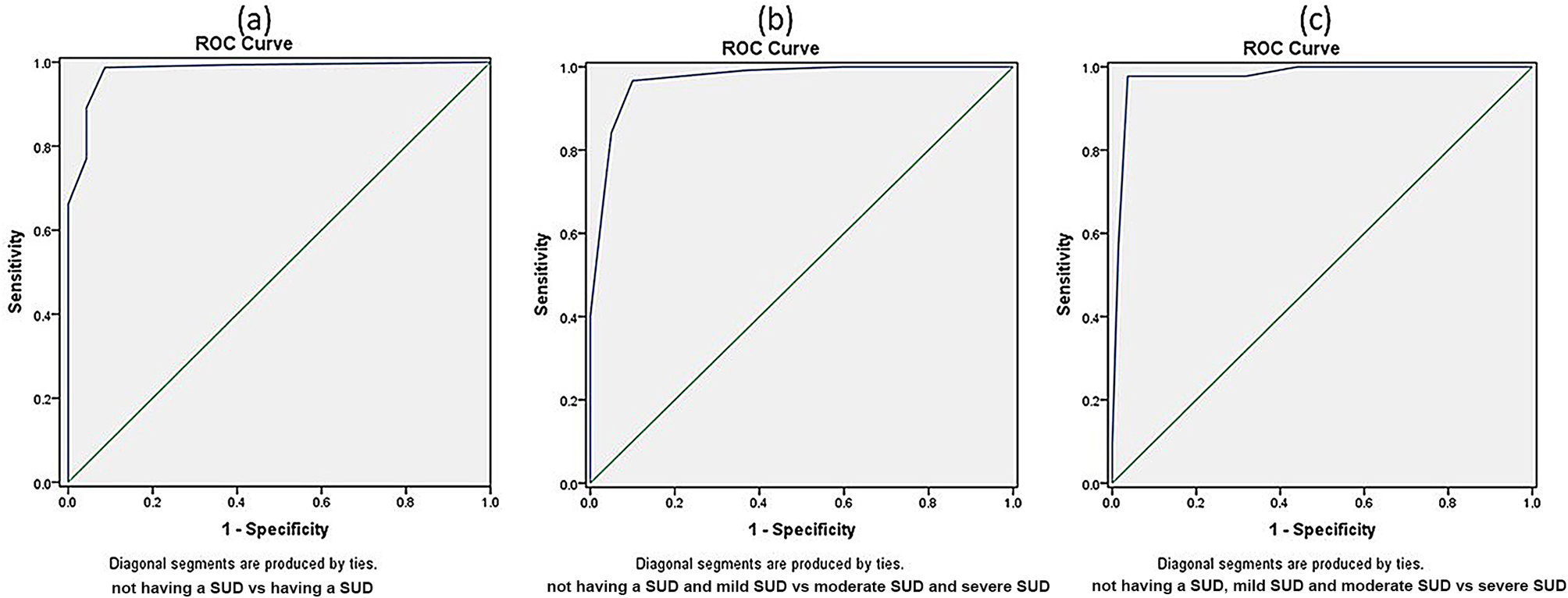
Receiver Operating Characteristic (ROC) curves for the Sinhala version of the Drug Abuse Screening Test. *SUD = substance use disorder

A clinical interview by the psychiatrist revealed not having a SUD among 14.7%, mild level SUD among 26.8%, moderate level SUD among 33.9% and severe level SUD among 24.6% of the participants. The developed ROC curves are shown in Fig. 3, and the coordinates of the developed ROC curves are presented in Table 4.

**Table 4:** Coordinates of the receiver operating characteristic (ROC) curves for each cut-off of DAST-SL

First cut-off				Second cut-off				Third cut-off			
Cut off	Sensitivity	1-specificity	Youden index	Cut off	Sensitivity	1-specificity	Youden index	Cut off	Sensitivity	1-specificity	Youden index
≥ 0.5	1.000	0.375	0.625	≥ 0.5	1.000	0.750	0.250	≥ 0.5	1.000	0.889	0.111
≥ 1.5*	**0.987**	**0.083**	**0.904**	≥ 1.5	1.000	0.600	0.400	≥ 1.5	1.000	0.822	0.178
≥ 2.5	0.897	0.042	0.855	≥ 2.5	0.992	0.367	0.625	≥ 2.5	1.000	0.711	0.289
≥ 3.5	0.776	0.042	0.734	≥ 3.5*	**0.967**	**0.100**	**0.867**	≥ 3.5	1.000	0.570	0.430
≥ 4.5	0.667	0.000	0.667	≥ 4.5	0.842	0.050	0.792	≥ 4.5	1.000	0.437	0.563
≥ 5.5	0.551	0.000	0.551	≥ 5.5	0.700	0.033	0.700	≥ 5.5	0.978	0.312	0.666
≥ 6.5	0.308	0.000	0.308	≥ 6.5	0.400	0.000	0.400	≥ 6.5*	**0.978**	**0.036**	**0.942**
≥ 7.5	0.173	0.000	0.173	≥ 7.5	0.225	0.000	0.225	≥ 7.5	0.556	0.015	0.541
≥ 8.5	0.026	0.000	0.026	≥ 8.5	0.033	0.000	0.033	≥ 8.5	0.089	0.000	0.089

* Optimal cut-off value

Youden Index(J) = Sensitivity+ Specificity -1

A cut-off value of ≥ 2 in the DAST-SL questionnaire demonstrated the presence of SUD (Table 4), with a sensitivity of 98.7%, specificity of 91.7%, positive predictive value (PPV) of 98.8% and negative predictive value (NPV) of 91.3% (Additional file 4). The area under the curve (AUC) of the ROC curve was 0.98, which was statistically different from the diagonal reference line (Fig. 3 [a]). According to the results, people who use drugs who were diagnosed as not having a substance use disorder (total score ≤ 1) were considered to have a low level of problems associated with psychoactive drug use (Table 4). A moderate level of problem severity had total scores of 2–3 (Table 4). The substantial level of problems was from total scores of 4–6. A cut-off score of ≥ 4 or more demonstrated a sensitivity of 96.7%, specificity of 90.0%, PPV of 95.2% and NPV of 93.2% (Additional File 4). The AUC for the cut-off value of ≥ 4 was 0.97, which is demonstrated in Fig. 3 [b]. Severe levels had DAST-SL total scores of ≥ 7 (Table 4). The cut-off score of ≥ 7 demonstrated high sensitivity (97.8%), specificity (96.4%), PPV (99.2%) and NPV (93.2%) (Additional file 4). The AUC for the cut-off value of ≥ 7 was 0.99 as demonstrated in Fig. 3[c]. The likelihood ratios for the cut-off values for moderate, substantial and severe degrees of drug problems are presented in Additional file 4.

### Reliability

The KR-20 test for the internal consistency of the DAST-SL questionnaire was 0.80 which is considered sufficiently reliable [47]. The test re-test reliability, which measures the stability of the tool over time, was also high [46]. The nine-item DAST-SL demonstrated an inter-class correlation coefficient of 0.97 (95% confidence interval 0.92–0.99). The DAST -SL scores recorded for the 20 psychoactive drug users at two different points in time are shown in Additional file 5.

## Discussion

The availability of a valid and reliable method to screen people who use drugs for their severity of psychoactive drug use is a timely need in Sri Lanka. While different agencies have developed many evidence-based tools for assessing psychoactive drug use severity [48] we aimed to select the most suitable tool that can be used in community settings or busy clinics, with or without medical personnel requiring specific training [15]. The DAST proved to be an ideal instrument due to its short length, ease of administration and comprehensiveness.

In this study, the item-to-subject ratio was taken as 1:20. Larger observations per item are known to be more suitable for ensuring stable results [35]. In addition, the literature reveals that a minimum sample size of 100 is adequate to achieve a good level of agreement for models with binary variables with one or two factors [49]. Therefore, the selected sample sizes were adequate for assessing construct validity. Even though the original item structure was changed, and only nine items were retained during cultural adaptation, the results of the EFA and CFA suggested that the modified Sinhala version of the DAST maintained its unidimensional structure. The model fit indices of the DAST-SL revealed a good fit. Only the Standardized Root Mean Square Residual value (SRMR) was slightly higher than the accepted value. However, as the SRMR does not perform well with binary data, it was considered acceptable [38].

During the CFA, the third item, “Have you ever had blackouts or flashbacks as a result of psychoactive drug use?” had the lowest correlation with the total scores. During the translation process, the wording of the items was not explicitly replaced by that of their Sinhala counterparts but was also paraphrased to make the definitions understandable and meaningful to the respondents. However, during the formative assessment and in-depth interviews for a different component of the study, it was evident that many people who use drugs lacked an understanding of their psychoactive drug use problems. It is more likely that they did not understand that the “flashbacks” and “blackouts” were due to psychoactive drug use because psychoactive drug use problems are not addressed openly in Sri Lanka.

When assessing the criterion validity of the DAST-SL, a clinical diagnosis provided by a psychiatrist was used. As no other method can ever replace clinical evaluation when making a psychiatric diagnosis [50], this approach was an added benefit to the accuracy of the tool. The cut-off value for SUD in the DAST-SL was two, which was high in sensitivity and specificity with good predictive values. Moreover, the cut-off values for different degrees of psychoactive drug use severity also demonstrated high sensitivity and specificity, proving it to be a valid screening tool.

The KR-20 test measures the internal consistency when the measurements of interest are measured at a dichotomous level [45]. The DAST-SL demonstrated a KR-20 value of 0.80 for the full scale. Which was considered as having adequate internal consistency [47]. The tool also demonstrated high test re-test reliability, indicating its internal validity. Thus, the modified version of the DAST demonstrates high reliability similar to those of the original version [25] and other cultural adaptations of the DAST tool [19, 20, 24, 28].

Screening for psychoactive drug use problems promotes early intervention. Early intervention not only stops or reduces harmful use but also improves health and social function, and reduces the risk of relapse [13]. The Sinhala version of the DAST is the first screening instrument in Sri Lanka that measures drug-related problems among people who use drugs. We hope that this tool will be utilised by different agencies working with people who use drugs in both the health and non-health sectors to improve their care. We hope the DAST-SL questionnaire will facilitate research on numerous themes linked to psychoactive drug use in Sri Lanka. Future studies can assess the prevalence of SUD among people who use drugs using the DAST-SL to determine treatment needs, identify gaps, and examine the connections between SUD and other health and social issues.

The DAST-SL, which was validated in the Sinhala language, was demonstrated to be a sound instrument for detecting substance use disorders among people who use drugs. However, this study was not without limitations. Our study used the RDS method for the recruitment of participants. Even though the recruitment quota was restricted to prevent clustering [35] (ensuring that recruitment is not biased by relying on a few individuals who are more successful recruiters) RDS enables the recruitment of peers with similar characteristics [35]. This may be the reason why all participants were able to speak and understand Sinhala well. The majority of the participants in the study sample were male. This may be due to the lesser number of females who use psychoactive drugs in the community than males in general, or due to the non-probability sampling technique used (RDS) that failed to reach females within the networks of the recruiters. In addition, the DAST-SL was not validated in the Tamil language and therefore cannot be used to screen people who use drugs who only understand Tamil. If future studies can validate the DAST-SL in the Tamil language, it would heighten its value.

## Conclusion

In conclusion, our study followed strict methodological standards outlined in the literature for translating assessment tools. The final modified Sinhala version of the DAST included nine items with good psychometric features that are consistent with those of the original scale and other language adaptations. Therefore, the DAST-SL was revealed to be a sound instrument that will enable screening for SUD and the severity of psychoactive drug problems in people who use drugs validly and reliably. Thus, we recommend that the DAST-SL, the Sinhala version of the DAST, be used by any organisation working with people who use drugs within or outside the health sector in Sri Lanka for screening purposes.

### Electronic supplementary material

Below is the link to the electronic supplementary material.

Supplementary Material 1

Supplementary Material 2

Supplementary Material 3

Supplementary Material 4

Supplementary Material 5

## Data Availability

The validated Sinhala version of the Drug Abuse Screening Test and datasets supporting the conclusions not included in the article are available upon reasonable request from the corresponding author through email.

## Abbreviations

SUD: Substance Use Disorder
DAST / DAST-SL: Drug Abuse Screening Test / Drug Abuse Screening Test-Sri Lanka (Sinhala version)
RDS: Respondent-driven sampling
EFA: Exploratory Factor Analysis
CFA: Confirmatory Factor Analysis
DSM-5: Diagnostic and Statistical Manual of Mental Disorders, fifth edition
SPSS software: Statistical Package for the Social Sciences software
ROC curve: Receiver Operating Characteristic curve
AUC: Area Under the Curve
PPV: Positive Predictive Value
NPV: Negative predictive value
KR-20: Kuder-Richardson Formula-20
ICR: Interquartile range
χ2: Chi-square
CFI: Comparative Fit Index
TLI: Tucker-Lewis Index
RMSEA: Root-Mean-Square Error of Approximation
SRMR: Standardized Root Mean Square Residual value
