# Supplementary figures and images for: Psychometric properties of the modified Drug Abuse Screening Test Sinhala version (DAST-SL): evaluation of reliability and validity in Sri Lanka
Source: BMC Public Health. 2024 Jul 3;24:1773. doi: 10.1186/s12889-024-19288-x (PMC11223402; doi:10.1186/s12889-024-19288-x)

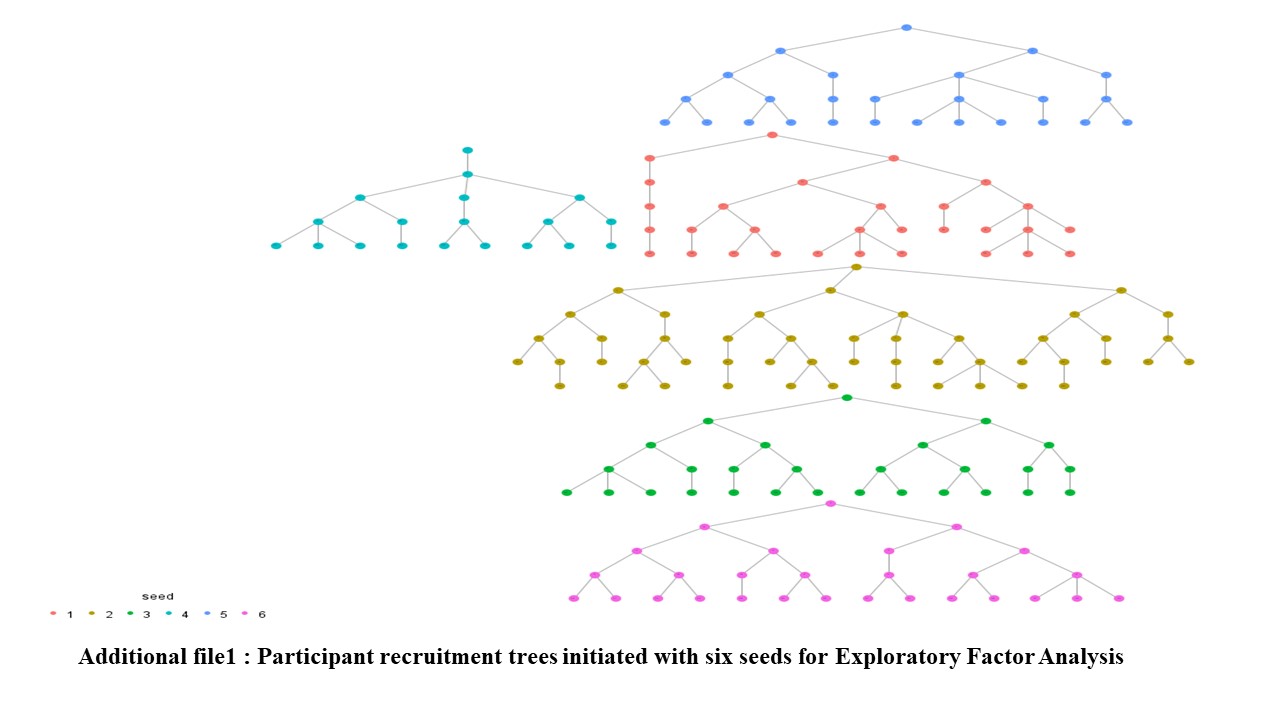

Supplement: Supplementary file 1 — Supplementary Material 1 [file 12889_2024_19288_MOESM1_ESM.jpg]

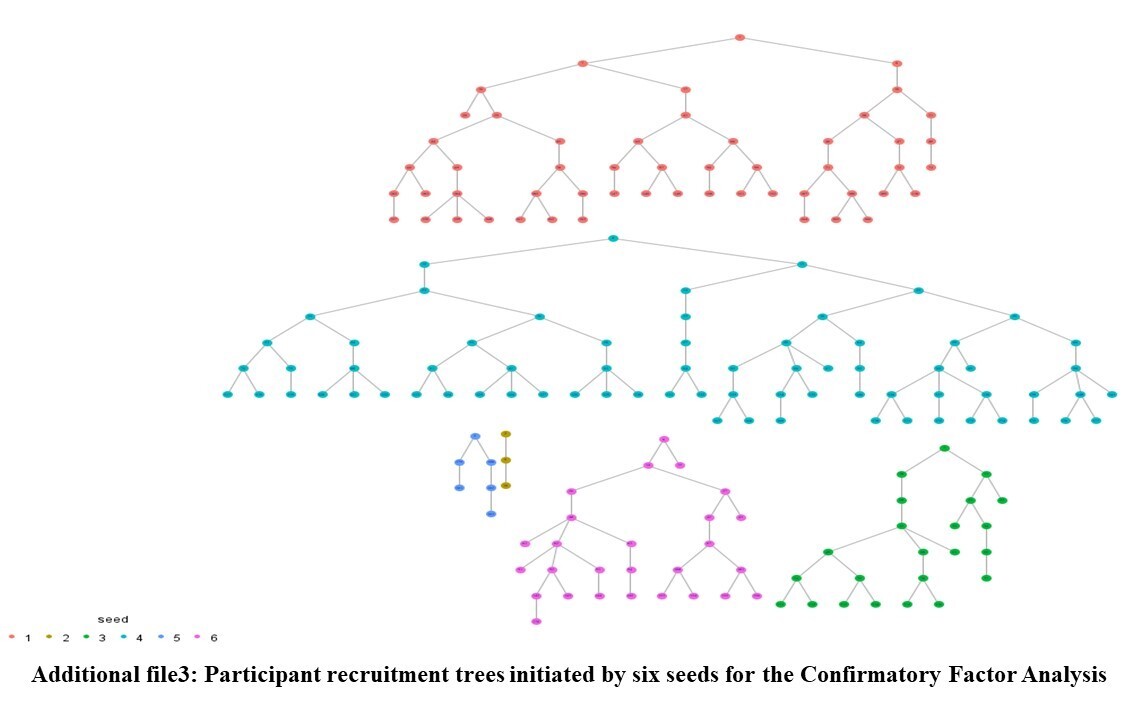

Supplement: Supplementary file 3 — Supplementary Material 3 [file 12889_2024_19288_MOESM3_ESM.jpg]
