# Supplementary material for: Psychometric properties of the modified Drug Abuse Screening Test Sinhala version (DAST-SL): evaluation of reliability and validity in Sri Lanka
Source: BMC Public Health. 2024 Jul 3;24:1773. doi: 10.1186/s12889-024-19288-x (PMC11223402; doi:10.1186/s12889-024-19288-x)
Supplement: Supplementary file 2 — Supplementary Material 2 [file 12889_2024_19288_MOESM2_ESM.pdf]

Additional File 1

Coupon no:

Questionnaire for the validation of Drug Abuse Screening Test – Sri Lanka (DAST-SL)

❖ **Network Questions (used to generate unbiased population estimates in Respondent Driven Sampling)**

I. How many people do you know in the Kandy District (they know you by name and you know theirs), who uses psychoactive drugs?

II. Out of those people, how many do you meet during a month?

❖ **Core questionnaire**

Part 1: Socio-demographic characteristics and drug use patterns

Please mark the correct answer with a cross (X)

1.1 Age (in complete years)

1.2 Gender i. Male ii. Female iii. Do not wish to say

1.3 Ethnicity

I. Sinhalese

i. Tamil

ii. Muslim

iii. Burger

iv. Other(please specify)……………………………

1.4 Highest level of education

i. Never attended school

ii. Grade 1-5

iii. Grade 6-10

iv. Passed General Certificate of Education (GCE) Ordinary Level

v. Grade 12-13

vi. General Certificate of Education (GCE)/Advanced Level and above

1.5 Current employment

i. Unemployed ☐

ii. Employed ☐

1.6 Monthly income

i. < Rs.20,000 ☐

ii. Rs. 20,001 – 40,000 ☐

iii. Rs. 40,001 – 60,000 ☐

iv. Rs. 60,001 – 80,000 ☐

v. Rs. 80,001 – 100,000 ☐

vi. >Rs. 100,001 ☐

1.7 Type of drug used (Can have more than one answer)

i. Cannabis ☐

ii. Heroin ☐

iii. Pregabalin ☐

iv. Tramadol ☐

v. Methamphetamine ☐

vi. Diazepam ☐

vii. Others (please specify) ☐

1.8 Frequency of drug use

i. 2 to 3 times a month or less ☐

ii. About once a week ☐

iii. 2 to 3 times a week ☐

iv. About once a day ☐

v. 2 times or more in a day ☐

Part 2: The modified Drug Abuse Screening Test Questionnaire (DAST-SL)

The following questions concern information about your possible involvement with drugs ***not including alcoholic beverages*** during the past 12 months.

"abuse" in question 1, refers to the use of prescribed or over-the-counter drugs in excess of the directions, and any nonmedical use of psychoactive drugs.
The various classes of drugs may include cannabis (marijuana, hashish), solvents (e.g., paint thinner), tranquilizers (e.g., Valium), barbiturates, cocaine, stimulants (e.g., speed), hallucinogens (e.g., LSD) or narcotics (e.g., heroin).

Please answer every question. If you have difficulty with a statement, then choose the response that is mostly right. Please mark your response with a (x) in the 'Yes' or 'No' column.

Item	Question	Yes	No
I.	Do you abuse more than one drug at a time?		
II.	Are you always able to stop using drugs when you want to?		
III.	Have you had "blackouts" or "flashbacks" as a result of drug use?		
IV.	Do you ever feel bad or guilty about your drug use?		
V.	Does your spouse (or parents) ever complain about your involvement with drugs?		
VI.	Have you neglected your family because of your use of drugs?		
VII.	Have you engaged in illegal activities in order to obtain drugs? (e.g. theft, fraud, prostitution)		
VIII.	Have you ever experienced withdrawal symptoms (felt sick) when you stopped taking drugs? (e.g. headaches, dizziness, chest tightness, difficulty breathing, nausea, vomiting, diarrhoea, stomach aches, tremors, muscle aches, sweating)		
IX.	Have you had medical problems as a result of your drug use (e.g. memory loss, hepatitis, convulsions, bleeding, etc.)?		

End of Questionnaire

Thank you!
