# Supplementary material for: Psychometric properties of the modified Drug Abuse Screening Test Sinhala version (DAST-SL): evaluation of reliability and validity in Sri Lanka
Source: BMC Public Health. 2024 Jul 3;24:1773. doi: 10.1186/s12889-024-19288-x (PMC11223402; doi:10.1186/s12889-024-19288-x)
Supplement: Supplementary file 4 — Supplementary Material 4 [file 12889_2024_19288_MOESM4_ESM.docx]

| **Validity Indicator** | Sensitivity, specificity, predictive values and likelihood ratios of the DAST-SL scores for the detection of drug use severity | | |
| --- | --- | --- | --- |
|  | **First cut-off**  **(≥2)**  (Moderate degree) | **Second cut-off**  **(≥4)**  (Substantial degree) | **Third cut-off (≥7)**  (Severe degree) |
| Sensitivity | 98.7% | 96.7% | 97.8% |
| Specificity | 91.7% | 90.0% | 96.4% |
| Positive Predictive Value (PPV) | 98.7% | 95.2% | 99.2% |
| Negative Predictive Value (NPV) | 91.3% | 93.2% | 93.2% |
| Positive Likelihood Ratio (LR+) | 11.85 | 9.67 | 27.2 |
| Negative Likelihood Ratio (LR-) | 0.01 | 0.03 | 0.02 |

**Additional File 4: Summary of validity indicators for different cut-off values of Drug Abuse Screening Test Sinhala Version (DAST-SL)**
